# In Vitro Evaluation of Antimicrobial Activity of Alimentary Canal Extracts from the Red Palm Weevil,* Rhynchophorus ferrugineus* Olivier Larvae

**DOI:** 10.1155/2017/8564601

**Published:** 2017-05-22

**Authors:** Gamal H. Sewify, Hanan M. Hamada, Hani A. Alhadrami

**Affiliations:** ^1^Deanship of Scientific Research, King Abdulaziz University, P.O. Box 80230, Jeddah 21589, Saudi Arabia; ^2^Department of Economic Entomology and Pesticides, Faculty of Agriculture, Cairo University, Giza, Egypt; ^3^Faculty of Applied Medical Sciences, Department of Medical Laboratory Technology, King Abdulaziz University, P.O. Box 80402, Jeddah 21589, Saudi Arabia

## Abstract

The invasive red palm weevil,* Rhynchophorus ferrugineus *Olivier (Coleoptera: Curculionidae), is considered one of the world's most devastating insect pests to palm trees. It was observed that larvae of this pest are able to inhibit microbial growth on the rearing media when they start feeding and this observation has led us to study the effect of red palm weevils on various microbial species. The antimicrobial effect of extracts from different parts of the alimentary canal on Gram positive bacteria (*Enterococcus faecalis *and* Staphylococcus aureus*), Gram negative bacteria (*Escherichia coli* and* Klebsiella *spp.),* Candida albicans,* and* Penicillium* sp. was tested using the agar well diffusion method. All extracts inhibited the tested microbial species. Foregut extracts had the greatest zones of growth inhibition.* Enterococcus faecalis*,* Staphylococcus aureus, *and* Penicillium *sp. were significantly sensitive to the extracts and had the largest growth inhibition zones. It is concluded that the gut extracts contain potent antimicrobial activity and may provide a new source of antimicrobial peptides.

## 1. Introduction

The invasive red palm weevil,* Rhynchophorus ferrugineus *Olivier (Coleoptera: Curculionidae), is considered the world's most devastating insect pest to palm trees. Since it was discovered in the Kingdom of Saudi Arabia and Egypt in 1987 and 1992, respectively [1], it has spread very rapidly. Female adults insert their eggs into palm tissues; hatched larvae feed within the trunk; and this behavior weakens infested palm trees and frequently kills them. As larvae feed, they make frass (a result of chewed up plant fiber), which mixes with plant sap, to fill cavities and tunnels formed by larvae. Several microbial agents including bacteria, actinomycetes, and fungi were isolated from the natural habitat of the red palm weevil [2, 3]. Mazza et al. [4] investigated the antimicrobial activity of the eggs and integument of both adult and larval stages of the red palm weevil. They found that the polar surface fraction of cuticle extracts inhibited the growth of Gram positive bacteria and the entomopathogenic fungus,* Beauveria bassiana*. This extract fraction had no effect on* E. coli *or* Metarhizium anisopliae *growth. On the other hand, the haemolymph of larvae and surface extracts of both small larvae and eggs did not inhibit the growth of any tested microorganism. During mass rearing of red palm weevils on sugar cane, Mazza et al. [4] noted that the microbial species that usually grow on sugar cane disappeared once the larvae emerged. They suggested that larvae of this pest have the ability to inhibit microbial growth on the rearing media when they start feeding. This observation has led us to study the effect of red palm weevils on various microbial species. Abdally et al. [5] detected the presence of certain immunity substances (lectins) in the midgut of the red palm weevil larvae and adults and studied their activities against mammalian erythrocytes. These lectins are believed to play a role in insect immunity as they mediate first line defense against invading pathogenic organisms. It is believed that these lectins have antimicrobial effect and can inhibit the growth and colonization of bacteria [6]. Throughout history, insects, their organs, and their secretions have been used as alternative medicines. Some of insects' components are still used to treat many diseases. Aqueous, alcoholic, and serratia peptidase-treated extracts from blow fly maggot (*Chrysomya rufifacies *[Macquart]) and housefly (*Musca domestica *Linnaeus) larvae were used to treat resistant infections and colon cancer, respectively [7, 8]. Also, venom and royal jelly taken from the honeybee* Apis mellifera *Linnaeus which are rich in antimicrobial peptides were used to treat arthritis, tumors, and leukemia [9, 10]. Living organisms are exposed to bacterial, fungal, and viral pathogens through direct contact, ingestion, and inhalation. The first line of defense of insects against these pathogens is the cuticle but once this physical barrier is penetrated their survival depends on the defense provided by the immune system, which has various humoral and cellular components [11]. The innate immune system is the first internal line of defense against these invaders and the acquired immune responses then follow [12]. Antimicrobial peptides (AMPs) appear to be an important and powerful constituent of the innate immune system. Both prokaryotic and eukaryotic organisms like plants, invertebrates, and vertebrates rely on AMPs as part of their defense against pathogens [13]. In insects, most of the known antimicrobial substances are comprised of peptides. These AMPs are produced from either the fat bodies or the haemocytes into the haemolymph [14]. Secretion of these peptides from the fat bodies into the haemolymph is considered the dominant mechanism in injured or infected insects. Insect epithelia, mainly the gut, salivary glands, and venom glands, also secrete tissue-specific AMPs [15, 16]. These gene-encoded AMPs are activated immediately after infection and act against a broad spectrum of microbes. These peptides kill bacteria (including strains resistant to conventional antibiotics), fungi, enveloped viruses, and even tumors. These properties make them excellent candidates for therapeutic agents [17]. Most of these AMPs are short proteins (<10 kDa), hydrophobic, and associated with membranes. The 3D structure of these peptides was discovered using nuclear magnetic resonance (NMR) technique and accordingly they were classified into *α*-helical AMPs, cysteine rich AMPs, *β*-sheet AMPs, AMPs rich in regular amino acids and AMPs with rare modified amino acids [18]. Many of these AMPs were originally isolated from insects [19].

The aim of this research is to investigate the antimicrobial effect of larval gut extracts on different types of Gram positive bacteria, Gram negative bacteria,* Candida albicans, *and* Penicillium *sp.

## 2. Materials and Methods

### 2.1. Sample Collection and Dissection

Red palm weevil larvae were collected from infested palm trees in the Ismailia Governorate (approximately 100 km east of Cairo). Large larvae (over 3 cm long) were used in the experiments. In the laboratory, thirty larvae were used to collect each part of the tissues included in the experiment (total gut, foregut, midgut, and hindgut). The larvae were immobilized on ice, sterilized using 70% ethanol, and then dissected in an ice-cold saline buffer under a stereoscopic microscope using sterile tools. The dissected tissues were removed, washed thoroughly in the saline buffer and transferred separately in sterilized tubes to a −20°C freezer for further use [20, 21]. The dissected tissues were homogenized separately using a hand-held glass grinder in a known volume of cold double-distilled water. Homogenates were then centrifuged at 10,000 ×g for 10 min at 4°C. The supernatant was collected in sterilized Eppendorf tubes and kept at −80°C until further use [21, 22].

### 2.2. Bacterial and Fungal Species

Four species of pathogenic bacteria from human origin (*Escherichia coli*,* Klebsiella *spp.,* Enterococcus faecalis*, and* Staphylococcus aureus*), in addition to the fungi,* Candida albicans *and* Penicillium *sp., were used for in vitro evaluation of the antimicrobial activity of these gut extracts. All tested microorganisms were supplied by the Microbiology Department, Faculty of Medicine, Cairo University.

### 2.3. Evaluation of Antimicrobial Activity

Microbial growth inhibition was assayed using the agar well diffusion method according to Magaldi et al. [23] and Valgas et al. [24]. Bacterial or fungal inoculum was uniformly spread all over the surface of the air-dried sterile agar Petri dishes using a sterile cotton swab. The agar was punched aseptically using a sterile tip to make a hole 5 mm in diameter. Twenty microliters of the gut extract were added to each well. Four replicates were used for each treatment and control wells were filled with 20 *μ*l of double-distilled water. Plates were incubated for 96 h under aerobic conditions and a suitable temperature for each tested microorganism (37°C for bacterial species and* C. albicans *and 25°C for* Penicillium *sp.). Antimicrobial activity was indicated by a clear zone in the agar (i.e., no microbial growth), which was measured in mm at 24, 48, 72, and 96 h after treatment. This test was repeated three times.

### 2.4. Statistical Analysis

A randomize complete design with three factors was used to analyze all data with four replicates. Treatment means were compared using a least significant difference (LSD) test as given by Snedecor and Cochran [25] using the ASSISTAT program [26]. A single factor ANOVA test was used to study the effect of each extract on different microbes and the significant differences among the inhibition zones for the foregut extract treatment. The correlation between time of exposure and the size of the growth inhibition zone was also studied.

## 3. Results

**Figure 1:**
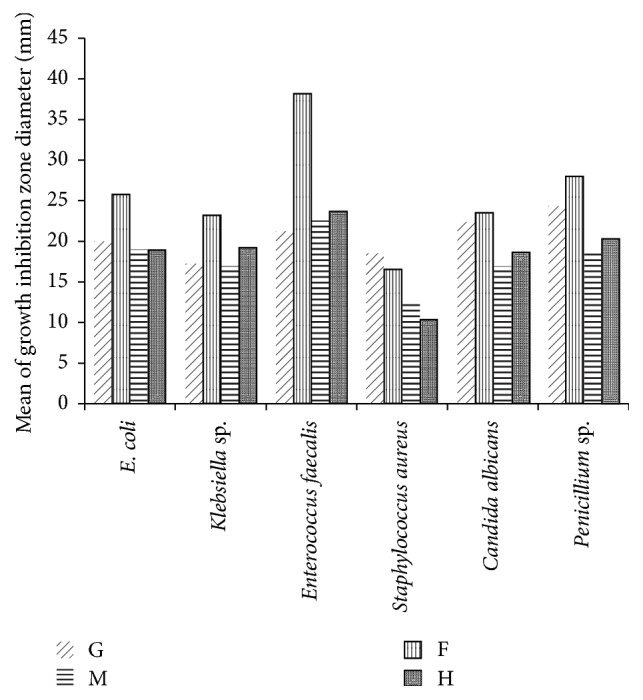
Effect of red palm weevil alimentary canal extracts on different microbial species 96 h after treatment. G: whole gut, F: foregut, M: midgut, and H: hindgut.

Antimicrobial activity of alimentary canal extracts from red palm weevil larvae was studied against* E. coli*,* Klebsiella *sp.,* E. faecalis*,* S. aureus, C. albicans, *and* Penicillium sp*. Results shown in Table 1 and illustrated in Figure 1 indicated that all extracts had an obvious inhibitory action against all tested microbial species. Statistical analysis using multivariable ANOVA showed that there were significant differences in the sensitivity of all microbes towards these extracts. The size of the inhibition zone for all treated microbes was directly proportional to the exposure time up till 96 hours. The means of inhibition zones presented in Table 1 showed that the foregut extract was the most effective extract on all microbes and the hindgut was the least effective. Gram positive bacteria and fungi were more affected by the treatment. At 96 h, the inhibition zone for* E. faecalis*,* S. aureus*, and* Penicillium *sp. was 38.25, 33.00, and 28.00 mm respectively. Gram negative bacteria were less affected and the inhibition zones were 25.75 and 23.25 mm for* E. coli* and* Klebsiella *sp., respectively.  Table 2 showed the correlation between time of exposure of all tested microbes to the foregut extract and the size of the inhibition growth zone. The results showed that the correlation coefficients were close to (+1) indicating a strong direct linear relationship between the inhibition zone size caused by the foregut extract and the exposure time for all microbes.

In Table 3, the presented data showed that there were significant differences among the growth inhibition zones of all tested microbes treated by different extracts after 96 h of the treatment.

The mean values of the inhibition zones in Table 1 showed that the foregut extract caused the largest inhibition zone for the tested microbial species. A single factor ANOVA was used to test the significance of difference in the inhibition zone size for the studied microbes. The results shown in Table 4 indicated that the foregut extract caused significant difference in the size of growth inhibition zone for all microbes.

## 4. Discussion

The agar well diffusion method clearly demonstrated that alimentary canal extracts of the red palm weevil used in this study had antimicrobial activity against all tested species of microorganisms. Furthermore, significant differences were observed among those activities. Gram positive bacteria, namely,* Enterococcus faecalis *and* Staphylococcus aureus*, followed by the fungus,* Penicillium *sp., were more sensitive and had considerably larger zones of growth inhibition after 96 h of the treatment with the foregut extract.* E. faecalis* recorded an inhibition growth zone of 38.25 mm;* Staphylococcus aureus* was 33.00 mm (Table 4). On the other hand, Gram negative bacteria were less responsive to the treatments and their inhibition zone after 96 h was 25.75 mm for* E. coli* and 23.25 mm for* Klebsiella *sp. The sensitivity of* Enterococcus faecalis *and* Staphylococcus aureus* can be attributed to the fact that Gram positive bacteria are monoderms surrounded by a cytoplasmic lipid membrane and lack the presence of the outer cell membrane which is present only in Gram negative bacteria. The absence of this outer membrane makes the bacteria vulnerable to the effect of AMPs of the gut extract. This argument is supported by many studies concerning Gram positive and Gram negative bacteria and their resistance to antibiotics [27, 28]. The outer cell membrane present in Gram negative bacteria is thought to play an important role as a protective mechanism against antimicrobial agents and antibiotic selection pressure. Also, some species of Gram negative bacteria like* Klebsiella *sp. are encapsulated in prominent polysaccharide capsules which give them more resistance and protect them from being killed by the antibiotic [29]. These facts support the results presented in this study where Gram negative bacteria had smaller inhibition growth zone and especially* Klebsiella *sp.

Turillazzi et al. [30] reported that the antibacterial activity in larval salivary secretions of* Polistes dominulu*s inhibits growth of Gram positive* Bacillus subtilis *and Gram negative* E. coli*. Since then, these insects were considered one of the main sources of antimicrobial peptides which have broad spectrum activity against bacteria, fungi, some parasites, and viruses. This experimental work proved for the first time that the alimentary canal of the red palm weevil larvae had antimicrobial activity. The results showed that treatment with foregut extracts caused the largest inhibition zones. The effect of salivary gland secretion might have a role in the antimicrobial activity of the foregut extract of the red palm weevil but this point needs to be furthermore investigated. It is also thought that the diet (i.e., palm fibers) might play a role in the distinguished effect of the red palm larval foregut extract. This hypothesis is supported by the fact that insects are known to collect useful substances from their diet and their surrounding environment [31]. For example, the* Vespa simillima* wasps build their nests from plant cellulose, which is collected from various plant sources. The 7,8-seco-para-ferruginone (SPF) found in* V. simillima *nests was also isolated from* Salvia prionitis *root and Japanese cedar bark; it possess antimicrobial activity against* S. aureus*,* Micrococcus luteus, *and* Alternaria alternate *[32]. Wasps collect these compounds from plant bark, which is used in the construction of their nests. Honeybees and stingless bees gather plant resins from trees and mix them with wax and glandular secretions to form propolis which is used to protect their colony from fungi, bacteria, and virus infections. In addition, feral honey bees coat the inside of their nests with resin to provide protection against pathogenic microorganisms [33].

Further studies are required to find out whether this antimicrobial activity originates from the gut cells and/or the lumen. The possibility that the secretion of salivary glands might enhance this activity remains to be elucidated. Thus, our biochemical analyses on different gut extracts are now in progress in an attempt to identify components responsible for this antimicrobial activity.

## 5. Conclusion

The study demonstrated the crude gut extracts of red palm weevil,* Rhynchophorus ferrugineus* Olivier larvae, exhibit potent antimicrobial activity against Gram positive bacteria and* Penicillium* sp. and could be explored as a potential source for antimicrobial peptides.

## Figures and Tables

**Table 1:** Antimicrobial activity of alimentary canal extracts from the red palm weevil larvae against different microbial species.

Exposure time (h)	Origin of extract	Growth Inhibition Zones (mm)						Mean
		Gram negative bacteria		Gram positive bacteria		Yeast	Fungus	
		*E. coli*	*Klebsiella *sp.	*E. faecalis*	*S. aureus*	*C. albicans*	*Penicillium *sp.	
24	G	11.75	10.75	13.25	10.75	13.50	15.00	12.50
	F	13.00	12.75	14.75	13.50	12.50	14.75	13.54
	M	10.75	11.50	11.50	13.50	12.50	12.50	12.04
	H	**9.75**	11.25	13.75	10.50	12.25	12.75	11.71
	Mean	11.31	11.56	13.31	12.06	12.69	13.75	12.45^d^

48	G	15.75	12.00	15.25	12.25	16.75	19.25	15.21
	F	18.50	13.50	16.50	13.75	14.00	22.50	16.46
	M	13.50	12.50	13.75	14.50	14.00	14.50	13.79
	H	12.75	11.25	12.50	11.75	13.75	14.75	12.79
	Mean	15.13	12.31	14.50	13.06	14.63	17.75	14.56^c^

72	G	18.25	13.00	16.75	13.75	17.75	23.00	17.08
	F	20.75	15.00	22.25	17.00	17.75	25.50	19.71
	M	16.25	14.50	17.25	16.50	15.25	17.00	16.13
	H	14.00	13.00	16.00	14.25	15.50	18.75	15.25
	Mean	17.31	13.88	18.06	15.38	16.56	21.06	17.04^b^

96	G	20.00	17.25	21.25	27.50	22.25	24.50	22.13
	F	25.75	23.25	**38.25**	33.00	23.50	28.00	28.63
	M	19.00	17.25	22.50	25.00	17.25	18.50	19.92
	H	19.00	19.25	23.75	20.50	18.75	20.25	20.25
	Mean	20.94	19.25	26.44	26.50	20.44	22.81	22.73^a^

G		16.44	13.25	16.63	16.06	17.56	20.44	16.73^b^
F		19.50	16.13	22.94	19.31	16.94	22.69	19.58^a^
M		14.88	13.94	16.25	17.38	14.75	15.63	15.47^c^
H		13.88	13.69	16.50	14.25	15.06	16.63	15.00^c^
Mean		16.17^c^	14.25^d^	18.08^b^	16.75^c^	16.08^c^	18.84^a^	

Columns labeled with the same letter are not significantly different (*P* > 0.05); G: total gut, F: foregut, M: midgut, and H: hindgut; LSD value at 0.05: microbial species = 0.7156, origin of extract = 0.5843, and time = 0.5843.

**Table 2:** Correlation between time of exposure to foregut extract and inhibition zone size.

Microbial species	Linear correlation coefficient
*E. coli*	0.989
*Klebsiella *sp.	0.880
*E. faecalis*	0.920
*S. aureus*	0.870
*C. albicans*	0.968
*Penicillium *sp.	0.960

**Table 3:** The effect of different extracts on the growth inhibition zone of tested microbes after 96 hours of treatment.

Microbial species	Origin of extract	ANOVA of growth inhibition zone size				
		Mean	Variance	*F*_cr_	*F*	*P* value
*E. coli*	G	20.00	13.33	3.49	4.91	0.0188
	F	25.75	2.25			
	M	19.00	18.00			
	H	19.00	0.67			

*Klebsiella *sp.	G	17.25	0.917	3.49	6.98	0.0057
	F	23.25	2.250			
	M	17.25	0.250			
	H	19.25	14.92			

*E. faecalis*	G	21.25	16.92	3.49	25.38	1.75*E* − 5
	F	38.25	4.92			
	M	22.50	11.00			
	H	23.75	6.92			

*S. aureus*	G	27.50	0.33	3.49	93.14	1.4*E* − 8
	F	33.00	3.33			
	M	25.00	0.67			
	H	20.50	0.33			

*C. albicans*	G	22.25	1.58	3.49	7.71	0.0039
	F	23.50	5.67			
	M	17.25	4.25			
	H	18.75	6.25			

*Penicillium *sp.	G	24.50	11.00	3.49	7.66	0.0040
	F	28.00	12.67			
	M	18.50	13.67			
	H	20.25	0.92			

There is significant difference among the inhibition zone sizes caused by different extracts at significance level 5%.

**Table 4:** The effect of foregut extract on the growth inhibition zone of tested microbes after 96 hours of treatment.

Microbial species	ANOVA of growth inhibition zone size				
	Mean	Variance	*F*_cr_	*F*	*P* value
*E. coli*	25.75	2.25	2.852	24.797	5.24*E* − 7
*Klebsiella *sp.	23.25	2.25			
*E. faecalis*	38.25	4.92			
*S. aureus*	33.00	2.00			
*C. albicans*	23.50	5.67			
*Penicillium *sp.	28.00	12.67			
